# Effects of Strategy-Based Memory Training for Older Adults: Do Booster Sessions Prompt Long-Term Benefits?

**DOI:** 10.3390/brainsci13091301

**Published:** 2023-09-09

**Authors:** Erika Borella, Enrico Sella, Anna Simonetto, Nicola Bellorio, Graziana Lenti, Laurence Taconnat, Elena Carbone

**Affiliations:** 1 Department of General Psychology, University of Padova, 35131 Padova, Italy; erika.borella@unipd.it (E.B.); enrico.sella@unipd.it (E.S.);; 2 Université de Poitiers et Centre de Recherches sur la Cognition et l’Apprentissage, Université de Tours, UMR-CNRS 7295, 37000 Tours, France; laurence.taconnat@univ-tours.fr

**Keywords:** memory training, memory strategies, booster sessions, long-term benefits, older adults

## Abstract

This study examined the efficacy of a strategy-based memory training for older adults at short- and long-term with two (5- and 11 months) follow-ups. We also explored whether booster sessions (additional training before the first follow-up) facilitated the maintenance of benefits. Thirty-three older adults received a training based on the teaching of different effective memory strategies. One group completed three booster sessions before the 5 months follow-up. Training gains were examined using a word-list and a face–surname association recall tasks, and transfer effects with a grocery-word list (GL) recall task, a working memory (WM) measure, and a perceived memory functioning questionnaire. Training gains and transfer effects to the WM measure emerged and were maintained up to the second follow-up. No benefits for the GL and perceived memory functioning were found. The “boosted” group had only a slight advantage—in one of the transfer tasks—as shown by effect sizes. This pilot study confirms the efficacy of strategy-based memory training in supporting older adults’ memory performance up to 11 months since training completion. However, booster sessions seem not to make a clear difference in prompting long-lasting benefits. Training features capable of fostering generalized, prolonged effects are worth investigating.

## 1. Introduction

Strategy-based memory training is a commonly used intervention for enhancing older adults’ episodic memory performance through the teaching and practice of effective memory strategies (e.g., imagery, method of loci, associations, organization) [1]. Systematic reviews and meta-analyses have proven this training effective in providing short-term improvements in older adults’ performance on memory tasks similar to the ones trained, i.e., training gains [1]. Short-term benefits to memory tasks not directly trained during the intervention, as well as in perceived memory functioning, i.e., transfer effects, were also found [1,2]. Maintenance of benefits over time, which is one of the aims of cognitive interventions, was also examined in few studies that included follow-up assessments. Training gains and—to a lesser extent—transfer effects were found to be maintained in some of these few studies, but not in others. The extent to which strategy-based memory training could prompt long-lasting benefits is thus worth investigating [1,2].

One of the factors that could favor the maintenance of benefits provided by cognitive interventions for older adults is the inclusion of booster sessions, conducted between post-test and follow-up assessments. These additional training sessions have been suggested to prompt long-term benefits by reactivating the use of strategies previously learned and practiced, which generally tend to fade after training [3]. However, their role in favoring training maintenance effects, and thus, their benefits, are not clear. To date, few studies have included booster sessions after memory training (see Table S1). In the ACTIVE study [4,5], four booster sessions were delivered at 11 months and then again at 35 months (before the 1- and 3-year follow-up assessments) from the beginning of the initial memory training, in which memory strategies were taught (see Table S1). Though the program proved effective in prompting short- and long-term training gains in objective memory outcomes, booster sessions did not provide additional benefits in prolonging their maintenance. Two other training studies have included booster sessions; however, they proposed a memory intervention that combined the teaching of memory strategies with psychoeducational activities triggering metacognitive processes. One study [6] did not find maintenance effects and additional benefits of booster sessions, while the other [7] did (see Table S1). No clear conclusions can thus be drawn regarding the efficacy of including additional training (booster) sessions to prompt the maintenance of any benefits provided by memory training and, in particular, strategy-based ones.

The present double-blinded pilot study thus aimed to investigate the efficacy of a strategy-based memory training for typically aging older adults not only at short-term, but also at long-term. Therefore, we included two follow-up assessment sessions: one 5 months after ending the training (and after the booster session presentation to a group of participants only), and a second one 11 months later. To clarify whether providing additional training could foster long-lasting benefits by reactivating an efficient strategy use, the usefulness of booster sessions was also ascertained.

Toward these aims, participants took part in five weekly group sessions of a strategy-based memory training. We employed a training program used by professionals in the applied context [8], which was based on other effective strategy-based memory training procedures [9,10]. Because multiple training strategies has been suggested to favor training benefits [1], different effective encoding memory strategies to facilitate later retrieval were proposed. Mental imagery strategy was taught and practiced first, and then two other mnemonics based on applying mental imagery to encode information in a more structured manner were taught and practiced, that is, method of loci and face–surname association techniques [1,11]. We incorporated, in the program, other features considered to prompt the benefits of teaching memory strategies, also by indirectly triggering older adults’ engagement in effortful cognitive tasks [2], such as variations of task demands and length (training difficulty), group discussions and individual -optional- at-home additional practice. Moreover, because music listening favors engagement in the effortful cognitive activities [12], thereby holding the potential to maximize intervention benefits, a pre-training activity based on listening to a music excerpt was also adopted.

A group of participants was involved in three booster sessions given 4 months after the initial training completion (right before the 5-month follow-up) to receive repetition of and additional practice with the memory strategies learned in the memory training.

Training gains were assessed with memory tasks similar to the ones trained (a word list and a face–surname association recall task). Transfer effects were also assessed, including a more ecological long-term everyday memory task (a grocery-word list recall task). Due to the key role of working memory (WM) in complex cognition and its age-related decline [13], a WM measure was also presented. A measure of memory complaints (the Memory Complaint Questionnaire—MAC-Q—[14]) was included to ascertain any benefits in perceived memory functioning.

In line with previous evidence [1], we expected to find short-term training gains in memory tasks similar to the ones trained. Short-term transfer effects to the untrained long-term everyday memory measures were also expected [1,2]. According to the strategy mediation hypothesis, the use of effective strategies is associated with good WM performance [15], and previous findings [16] show that immediate improvements in the untrained WM task were also expected. Because practicing memory strategies may enhance awareness and control over one’s memory abilities [17], improvements in perceived memory functioning (MAC-Q) were expected, at least in the short-term. Based on previous initial findings [4,5,6,7], we also expected training gains and transfer effects to be long-lasting. We explored whether booster sessions could facilitate their maintenance at the first and second follow-up, before which no booster sessions were presented.

## 2. Materials and Methods

### 2.1. Participants

This study involved 33 older adults who are native Italian speakers (age range: 63–75 years), recruited from communities in collaboration with a cultural association in Northern Italy. Inclusion criteria were as follows: (a) good physical and mental health status, as assessed by a semi-structured interview [18]; (b) a Mini-Mental State Examination score [19] of 27 or higher (i.e., no signs of cognitive impairment or incipient dementia); and (c) an average score on a vocabulary subtest [20].

Participants were randomly assigned to the memory training+booster session group (TG1; *n* = 17; 13 females), which was involved in the strategy-based memory training and received three booster sessions 4 months after the posttest and before the first (5 months) follow-up, whereas the memory training group (TG2; *n* = 16; 12 females) were involved in the strategy-based memory training only. Based on a previous study that used a similar memory training with older adults [9], and which reported a medium-to-large range of Cohen *d* (ranging from 0.76 to 1.00) on the cognitive measures of interest, a power analysis (using the R software’s pwr library) showed that we would need an estimated number of participants ranging from 16 to 28. We involved 48 participants, but due to exclusion criteria, we had a final sample of 33 participants, which was still sufficient to achieve a power of 0.80 (*p* < 0.05). The two groups did not differ in terms of age, education, vocabulary, and gender distribution, χ² _(1)_ = 0.10, *p* = 0.92 (see Table 1).

The study was approved by the local university research ethics committee for psychological research (No. 4411).

### 2.2. Materials

#### 2.2.1. Training Gains

*Word list recall task* (WL). Participants were presented an audio-recorded list of 15 words (2 s per word), and they had to recall as many words from the list as they could. The dependent variable was the total number of correctly recalled words (max = 15).

*Face–surname associations recall task* (adapted from [10]). Participants were given eight colored photographs of faces with surnames displayed below them, each printed on a single page and provided in a fixed order. Participants were allowed to learn the face–surname associations for 2 min (study phase). Then, the photographs without the surnames were presented, and participants had to write down the surnames paired with each face (recall phase). The dependent variable was the total number of correctly recalled face–surname associations (max = 8).

#### 2.2.2. Transfer Effects

*Grocery-word list recall task* (GL; adapted from [10]). Participants were presented an audio-recorded list of 15 grocery items (2 s per item). After a delay of about 10 min (in which they completed the vocabulary subtest as a filler), they were asked to recall as many items from the list as they could. The dependent variable was the total number of correctly recalled items (max = 15).

*Listening Span Test* (LST; [18]). This task consisted of sets containing increasing numbers (2, 3, 4, 5, and 6) of simple sentences. Participants were instructed to listen to each sentence, judge its plausibility, and retain the last word of each sentence to recall it at the end of each set. The dependent variable was the total number of correctly recalled words, expressing a measure of WM performance (max = 20).

Parallel versions of these tasks (three for the WL, face–surname association, and GL tasks and two for the LST) were created and administered in a counterbalanced fashion across testing sessions.

*Memory Complaints Questionnaire* (MAC-Q; [14]). Participants were instructed to rate their ability to perform five distinct everyday activities involving memory functioning (e.g., remembering names, recalling telephone numbers, and remembering items to buy in a grocery store), as well as their general ability to carry out activities involving memory, as compared to when they were younger, using a Likert scale from 1 (much better now) to 5 (much worse now). The dependent variable was the sum of the scores on each item, with higher scores expressing worse subjective memory functioning (max = 35).

### 2.3. Procedure

All participants attended four individual sessions lasting about 60 min each, one for each of the pretest, posttest, 5-month, and 11-month follow-up assessments, to complete the battery of tasks and questionnaires for ascertaining the short and long-term training efficacy (see Table S2).

After the pretest, they took part in a group-based memory training, which consisted of five weekly group sessions (delivered to groups of 5–7 participants), lasting about 90 min each.

Each session followed the same structure (see Table S2). In particular, a 10 min introduction opened each session. The experimenter welcomed participants, summarized the content of the previous session (since Session 2) and introduced the session’s main theme.

The introduction was followed (since Session 2) by an 8 min pretraining activity, involving listening to a music excerpt. Participants chose between two music pieces (Mozart’s Sonata K 448 or Albinoni’s *Adagio in G minor*) and then listened to the chosen movement for about 8 min [12].

Then, participants underwent 45–50 min main session activities, which were dedicated to the teaching and practicing with different effective encoding memory strategies.

In particular, the first two sessions focused on the teaching and practicing with mental imagery, that is, mentally visualizing to-be-recalled information [21]. The third session was then entirely dedicated to the teaching and practicing with the method of loci, involving the use of a very well-known familiar route as a structure for encoding and retrieving new information which are successively associated, also thanks to mental imagery, with locations of the mental route [22]. The fourth session focused on the teaching and practicing with face–surname association techniques, that is, grasping distinctive and imageable features both in the face and in the surname, and mentally linking them with an interactive image [1,10]. The last session provided additional practice with both the method of loci and the face–surname association techniques (see Table S2).

Within each session, the training difficulty, in terms of cognitive effort of the tasks’ demands, was modulated by providing practical activities that progressively guided participants to familiarize, practice and flexibly use the strategy that was taught (see Table S2). Regarding imagery strategy, participants underwent structured tasks based on (i) creating and manipulating simple mental images, and (ii) mentally visualizing and interrelating different stimuli into an integrated visual representation (interactive images). Participants were encouraged to pay particular attention to the vividness (i.e., clarity, brightness, intensity) of the images created, as it favors the encoding and subsequent retrieval of information [23], thereby making the imagery strategy more effective. Then, they were presented with a series of word list recall tasks that varied in terms of number of stimuli to be recalled (from 10 in the first session to 12 in the second) and demands, such as manipulating different features (e.g., dimension, movement) of the mental images created. In the second session, participants also learned, through familiarization activities, how to improve the vividness of the mental images created, including proprioceptive and sensorimotor features (e.g., visualize oneself interacting with the to-be-imaged stimuli [24]), and then practiced with word list recall tasks. For the method of loci, participants underwent a series of practical activities meant to guide them to progressively familiarize, practice and acquire each step needed to be successfully applied to the mnemonic. First, ad hoc activities were presented to allow them to learn their selected path/own route (e.g., chose a familiar outdoor route, select locations, mentally visualize the path and its locations forward and backward). Then, they learned how to use mental imagery (i.e., creating simple or interactive images) to allocate the information to be recalled within each location of their path. Finally, a series of activities requiring applying the mnemonic to remember word lists were proposed, varying the word list presentation modality (e.g., either the experimenter orally presented the word list, or it was written on a sheet of paper). Participants were guided to progressively familiarize, practice, and acquire each step needed to apply the face–surname association techniques: a series of activities dedicated to select and focus attention to distinctive facial features that make them memorable were presented first, followed by activities that taught them how to manipulate the meaning of the given surname to make it imageable. Then, face–surname association recall tasks were proposed to allow them to practice with the face–surname association techniques.

Finally, a 10 min conclusion ended each session. After a final group discussion on the memory strategy they had learned, the experimenter reminded participants about the next session appointment.

Group discussions were facilitated throughout the session (e.g., at the end of each practical activity and at the very end of the session) to promote reflections on the usefulness of the practiced memory strategies, ways to overcome possible challenges in applying them, and ways to employ them successfully in everyday life, benefitting from the participatory nature, vicarious experience, and social stimulation of the group setting [9].

Moreover, since Session 2, practical voluntary at-home activities were provided for participants to complete as additional individual practice on the acquired memory strategies to automatize their use and make them less taxing in everyday life (see Table S2). Although homework assignments were not mandatory, participants were invited to bring the activities performed at home to the following session, as is standard practice in these training programs (e.g., see [25,26,27]). At the beginning of the session, when a summary of the previous content was proposed, the experimenter dedicated time to discuss the homework assignments and invited participants to share them with the other group members. Such an approach allowed the experimenter not only to check whether participants actually performed homework assignments, but also to provide feedback on potential difficulties they were encountering or whether there were unclear aspects on the mnemonics taught that need clarification before moving forward with the training activities.

Participants in TG1 were involved in three additional weekly group-based booster sessions lasting about 90 min each, organized with the same structure as the initial training (i.e., introduction, pre-training activity, main session activities and conclusion). Each booster session aimed to provide a repetition and additional practice with the memory strategies acquired in the initial training. Therefore, during the first booster session, participants were reminded how to use the mental imagery strategy to learn and remember information successfully; then, they underwent a series of word list recall tasks to practice again (see Table S2). In the second booster session, the experimenter reminded participants how to use the method of loci to learn and remember information successfully. After rehearsing their own, familiar and learned route, which was used during the initial training, participants practiced again with the mnemonic, performing a series of word list recall tasks (see Table S2). In the third booster sessions, the experimenter reminded participants how to apply face–surname association techniques, and then participants were allowed to practice again with face recognition and face–surname association recall tasks (see Table S2). Participants received practical at-home activities to complete individually between sessions (see Table S2).

The assessment sessions were provided by a trained experimenter (a psychologist), who was blinded with respect to the participants’ allocation to the experimental conditions. The training sessions and the booster sessions were provided by a second trained experimenter (another psychologist).

### 2.4. Statistical Analyses

First, each group’s baseline performance was compared by means of separate analyses of variance (ANOVAs) on their pretest performance in all the outcome measures of interest, with group (TG1 vs. TG2) as the between-subjects factor.

Then, to ascertain training effects, a series of mixed-effect ANOVAs with group (TG1 vs. TG2) as the between-subject factor, and session as the repeated measures, separately—due to the sample size characteristics—for immediate (pre-test vs. post-test) and long-term benefits (pre-test vs. 5-month or 11-month follow-up), were run for each measure of interest.

To further examine differences between the two groups in terms of short- and long-term training gains and transfer effects, gain scores were also calculated for each outcome as follows: posttest–pretest performance for the short-term benefits and 5-month or 11-month follow-up, and pretest performance for the long-term benefits. Independent *t* tests were then run with group (TG1 vs. TG2) as the between-subjects factor, and gain scores as dependent variables.

As the literature recommends [28], Cohen’s *d* [29] were calculated separately for TG1 and TG2 to understand and descriptively compare the extent of training gains and transfer effects, both at the short-term (between pre-test and post-test) and the two long-term timepoints (between pre-test and the two follow-ups, respectively) of each group. This index, expressing the effect size of the comparisons, was used with the Hedges and Olkin [30] correction, as performed in the training literature, to avoid the small sample bias.

## 3. Results

The two groups did not differ in any measures at baseline (see Table 1).

As for training gains in the WL and the face–surname association recall task, results from mixed-effect ANOVAs showed a main effect of session, but not of group, at short-term (see Table 2), which is an improvement in participants’ performance from pre-test to post-test in these tasks (*Mdiff* = 0.96, *p* = 0.01; *Mdiff* = 1.03, *p* = 0.003; respectively), regardless of group.

A main effect of session, but neither of group nor group X session interaction, also emerged for the WL and the face–surname association recall task when the two follow-up timepoints were considered (see Table 2). Participants performed better in these tasks from pre-test to the 5 months follow-up (*Mdiff* = 1.45, *p* < 0.001; *Mdiff* = 1.45, *p* = 0.004; respectively), and from pre-test to the 11 months follow-up (*Mdiff* = 1.50, *p* < 0.001; *Mdiff* = 1.49, *p* < 0.001; respectively), regardless of group.

As for the transfer effect in the LST, a main effect of session, but not of group or group X session interaction, emerged at the short-term (see Table 2), with participants recalling more words from pre-test to post-test on this task (*Mdiff* = 1.63, *p* = 0.004).

A main effect of session, but neither of group nor group X session interaction, emerged for the LST when the two follow-ups were considered (see Table 2). Participants, regardless of group, recalled more words from pre-test to the 5 months follow-up (*Mdiff* = 1.58, *p* = 0.006), and from pre-test to the 11 months follow-up (*Mdiff* = 1.39, *p* = 0.026) in this task.

No significant effects at short- and long-term were found, instead, for the GL and the perceived memory functioning measures (see Table 2).

When gain scores were considered, results (see Table S3) confirmed the lack of differences between the two groups for any of the outcome measures considered, both for the immediate and the two maintained gain scores.

Concerning effect sizes, short-term, medium effect sizes (larger for TG1 than for TG2) emerged for training gains in the WL and the face–surname association tasks. From pre-test to the two follow-ups, effect sizes became large for both groups in these two tasks (see Table 2). For the LST transfer task, short-term medium effect sizes emerged in both groups. From pre-test to the 5 months follow-up, effect sizes became large for the TG1 and did not vary (medium) for the TG2.

From pre-test to the second (11 months) follow-up, medium effect sizes were found for both groups (see Table 2). For the other transfer task (the GL), TG1 had smaller effect sizes at the short-term, which became medium at the two follow-ups. For TG2, the effect sizes were medium at short-term, but became small or negligible at the two follow-ups (see Table 2). Effect sizes were overall small-to-negligible for the MAC-Q in both groups (see Table 2).

## 4. Discussion

Although the aim of cognitive interventions is to provide long-lasting benefits, few studies have examined the long-term effects of memory training among older adult population, and even less the impact of additional practice (booster sessions) to prompt them. The present study, which should be considered a pilot, is among the few in aging that presents two follow-ups, at 5 and 11 months from the training completion, to ascertain not only the short-term but also the long-term efficacy of a strategy-based memory training for typically aging older adults in improving performance in trained and untrained memory tasks, as well as in ameliorating perceived memory functioning. It also innovatively ascertains the effect of booster sessions, yielded to one of the trained groups immediately before the first follow-up assessment, to prompt the maintenance of training gains and transfer effects.

In line with previous evidence [1] and our expectations, our strategy-based memory training, as shown by ANOVAs, gain scores and medium (or close to medium) effect sizes proved effective in providing short-term training gains in memory tasks similar to the ones trained (i.e., the WL and face–surname association recall tasks). Moreover, as expected and in line with previous findings [16], short-term transfer effects to the WM measure emerged, along with medium effect sizes for this task in both groups, with no differences in their gain scores.

No benefits emerged; instead, they occurred for the other more ecological transfer task, i.e., the GL. Though such a result was unexpected, it is worth mentioning that the stimuli used in this task were mainly related to the same category—that is, food (e.g., vegetables, fruits)—an aspect that could have favored interference and affected recall performance [31]. The features of this task might thus explain the lack of effects for this outcome.

The lack of short-term benefits also in perceived memory functioning was in part unexpected too [17]. It could be that the survey chosen was not capable of capturing the benefits provided by the training regimen used here, based on teaching memory strategies. Factually, older adults are shown to benefit from memory training in terms of perceived memory functioning when also metacognitive/emotional-motivational processes are directly targeted by the intervention [2]. This was not conducted here and could have thus prevented our participants to gain ameliorated subjective experience of their memory functioning in everyday life.

Notably, slight differences in the benefits of the intervention provided between the two groups emerged in the post-test phase. Participants in both groups underwent the initial training with the same experimenter, in the same period, and with the same scheduling, and there were no substantial differences between groups in terms of compliance. In fact, all participants in both groups attended all the sessions, and the experimenter reported that most participants completed homework assignments consistently across training sessions and did not notice differences in their completion between the two groups. Therefore, this trend might be due to individual differences related to engagement or motivation toward performing the proposed training activities, which are known to be of potential influence on cognitive intervention’s efficacy, even among older adults [32,33]. However, this is only a speculation because we did not assess these aspects here. A closer monitoring of homework assignments would have also allowed us to understand better how participants were experiencing the training program. Therefore, future studies should consider these issues to explain differences in the training experiences across groups and, broadly speaking, who might have benefited—more or less—from the training.

When looking at long-term benefits, our results, as expected and in line with previous findings [4,5], revealed that training gains (in the WL and face–surname association recall tasks), as well as transfer effects to the WM measure (the LST), were long-lasting, both when the 5- and 11 months follow-ups timepoints were concerned.

Booster sessions did not really seem to play a role in prompting additional benefits in the longer term in terms of training gains, as suggested also by the large, comparable effect sizes found for both groups in the WL and the face–surname association recall tasks from pre-test to 5 and 11 months after the initial training completion. However, looking at transfer effects, the “boosted” group displayed a large effect size in the WM measure from pretest to the 5-month follow-up (i.e., after completing the booster session), which was medium for the latter. Intriguingly, such a TG1 “advantage” faded from pretest to the 11-month follow-up, with effect sizes in the LST becoming medium for both groups. Furthermore, effect sizes in the GL recall task were medium (i.e., larger than in the short-term) from pretest to the 5-month follow-up (i.e., after the booster sessions had been completed) for TG1, which was not the case for the participants who did not receive booster sessions, and TG1 maintained such an advantage up to the 11-month follow-up. Although such a pattern of results should be taken with caution and deserve to be further explored, it could suggest, albeit descriptively, that additional training sessions hold the potential to prompt improvements in the intended direction, i.e., reactivate, boost and prolong the benefits provided by learning and practicing with memory strategies when transfer effects are concerned, though they remain dependent on the outcome and the assessment timepoint considered.

Altogether, our results align with previous but limited evidence highlighting the efficacy of strategic memory training for older adults in promoting short- and long-lasting benefits in tasks similar to the ones trained [1,4,5]. They also extend previous evidence by showing the utility of such interventions in supporting, even in the longer term, a core cognitive mechanism like WM. Such a result is in line with the notion that learning effective memory strategies allows older adults to rely upon long-term retrieval structures, which facilitate the lowering of attentional demands of the WM task and later retrieval. The different allocation of resources to task goals prompted by the training seems to have increased the performance of WM, one the most sensitive mechanisms in aging largely involved in everyday life performance [13]. Short- and long-term benefits seem, however, to be confined to outcomes that do not imply other processes (e.g., interference/inhibition, metacognitive/emotional-motivational aspects) not directly trained/targeted, as was the case of the GL and the MAC-Q used here, an issue that deserves to be further explored. Notably, the challenge of finding transfer effects and their maintenance is well recognized [1,2], also in large-scale studies and even after the use of booster sessions [4,5].

The lack of clear evidence of a beneficial, additional role of booster sessions calls upon the need to explore further ways to design and schedule additional training to be more effective. It could be that the three booster sessions with a wealth of content and intensity, after 4 months from the initial training completion, were not enough for our participants to fully benefit from the additional training in the long run. Further studies are therefore needed to understand whether repeated additional booster sessions, spaced between the follow-up periods, would be a better option to provide plain, consistent, generalizable, and endurable benefits. In addition, it is worth mentioning that the one study [7] which found short- and long-term improvements in both objective and perceived memory outcomes, with additional benefits provided by booster sessions, incorporated the strategy-based training with activities aimed at ameliorating metacognitive/emotional-motivational processes, unlike ours and previous ones [4,5]. We included some features thought to favor the engagement in effortful cognitive activities as well as to trigger metacognitive (e.g., awareness, monitoring, and control) and motivational processes in our training regimen, such as the music listening pre-training activity, group reflections and discussions and individual practice. However, combining or incorporating the teaching of memory strategies with specific (psychoeducational) activities that directly and purposely target metacognitive/emotional-motivational processes, would have foster effects, also in terms of perceived memory functioning [2].

Notwithstanding these promising findings that confirmed the training program used here was effective [11], there are some limitations that deserve acknowledgment. Future research should replicate and expand the results of our pilot study with a larger sample size. By doing so, the role of individual characteristics, such as broad predispositions (e.g., personality traits, mindset) or metacognitive (e.g., beliefs and attitudes toward own cognitive functioning, self-efficacy) and motivational aspects, accounting for training benefits, could be further analyzed [32,33,34]. This issue was not considered here and is worth investigating, because it would allow gaining a better understanding of the efficacy of cognitive interventions among the older adult population, such as the strategy-based memory training used here, and the role of including additional training to prompt long-lasting benefits.

Including a control condition, scheduling individual phone contacts between one session and another to monitor participants’ compliance and engagement with the training program [25,27], and using a broad battery of tasks, also encompassing ecological measures, is warranted to further investigate the benefits of strategy-based memory training [1] and the mechanisms affecting them.

## 5. Conclusions

In conclusion, this study further confirms that strategy-based memory training can be considered a promising non-pharmacological approach to be used in clinical/applied contexts to support older adults’ memory performance not only in the short-term, but also in the long-term. Booster sessions do not seem to make a difference, despite their potential to reactivate learned strategies. A more systematic assessment of long-term effects of such interventions, as well as new ways to design and schedule booster sessions to clarify their usefulness towards prompting maintenance of training benefits, warrants further research efforts.

## Figures and Tables

**Table 1:** Descriptive statistics (means and standard deviations) of the socio-demographic characteristics, screening and outcome measures by group and assessment session, and results of the differences between the two groups at pre-test.

	Pre-Test		Groups’ Differences		Post-Test		5 Months Follow-Up		11 Months Follow-Up	
	TG1	TG2			TG1	TG2	TG1	TG2	TG1	TG2
	*M (SD)*	*M (SD)*	*F*_(1,31)_	*p*	*M (SD)*	*M (SD)*	*M (SD)*	*M (SD)*	*M (SD)*	*M (SD)*
Age (years)	67.47 (3.30)	68.00 (3.28)	<1							
Education (years)	15.59 (4.38)	15.56 (5.03)	<1							
Vocabulary	54.47 (12.55)	54.50 (10.15)	<1							
Word List	6.35 (1.53)	6.38 (1.14)	<1		7.59 (2.18)	7.06 (2.04)	7.82 (1.66)	7.93 (1.33)	8.00 (1.61)	7.85 (2.30)
Face–surname associations	4.75 (1.65)	4.50 (2.09)	<1		5.71 (1.89)	5.31 (1.74)	5.91 (1.92)	6.33 (1.54)	6.00 (1.34)	6.38 (1.71)
Grocery-word list	6.24 (2.41)	6.19 (2.07)	<1		7.00 (2.12)	7.31 (2.30)	7.91 (2.94)	6.33 (1.87)	7.64 (1.56)	6.38 (1.38)
Listening Span Test	8.94 (2.27)	8.50 (2.58)	<1		10.65 (2.64)	10.06 (1.98)	11.18 (2.18)	9.93 (1.94)	10.45 (1.69)	10.23 (2.20)
Memory complaint questionnaire	27.24 (2.70)	28.88 (3.05)	2.67	0.112	26.18 (3.69)	28.44 (4.91)	25.82 (3.34)	28.60 (4.79)	27.00 (2.14)	27.69 (4.42)

Notes. SD: standard deviations; TG1: memory training+booster sessions; TG2: memory training.

**Table 2:** Results from mixed-design ANOVAs for each outcome measure of interest, and effect sizes (Cohen’s d) for the short- and long-term benefits by group.

	Short-Term Benefits					Long-Term Benefits (5 Months)					Long-Term Benefits (11 Months)				
				TG1	TG2				TG1	TG2				TG1	TG2
	F_(1,31)_	*p*	η^2^_p_	*d*	*d*	F_(1,24)_	*p*	η^2^_p_	*d*	*d*	F_(1,22)_	*p*	η^2^_p_	*d*	*d*
Word list				0.64	0.41				0.90	1.22				1.02	0.82
Group	F < 1					F < 1					F < 1				
Session	7.23	0.011	0.19			15.53	0.001	0.393			16.94	<0.001	0.435		
Group × Session	F < 1					F < 1					F < 1				
Face–surname associations task				0.77	0.41				0.83	0.96				0.93	0.94
Group	F < 1					F < 1					F < 1				
Session	10.26	0.003	0.25			10.39	0.004	0.311			18.05	<0.001	0.462		
Group × Session	F < 1					F < 1					F < 1				
Grocery-word list				0.33	0.50				0.61	0.07				0.64	0.10
Group	F < 1					1.29	0.266	0.051			F < 1				
Session	3.95	0.056	0.11			1.38	0.251	0.054			1.50	0.233	0.064		
Group × Session	F < 1					1.68	0.207	0.065			2.65	0.118	0.108		
Listening Span Test				0.68	0.66				0.97	0.60				0.71	0.69
Group	F < 1					1.59	0.219	0.062			F < 1				
Session	9.62	0.004	0.24			8.94	0.006	0.271			5.74	0.026	0.207		
Group × Session	F < 1					F < 1					F < 1				
Memory complaint questionnaire				−0.31	−0.10				−0.46	0.07				−0.09	−0.31
Group	3.06	0.090	0.090			3.38	0.078	0.124			1.36	0.256	0.058		
Session	1.40	0.245	0.043			F < 1					F < 1				
Group × Session	F < 1					F < 1					F < 1				

Notes. TG1: memory training + booster sessions; TG2: memory training.

## Data Availability

The data that support the reported results will be made available by the authors upon reasonable request (elena.carbone@unipd.it).

## Supplementary Materials

The following supporting information can be downloaded at: https://www.mdpi.com/article/10.3390/brainsci13091301/s1, Table S1: Summary of studies on strategy-based memory training for older adults including booster sessions; Table S2: Description of the procedure and activities for the strategy-based memory training and the booster sessions; Table S3: Descriptive statistics for the short-term (post-test − pre-test) and long-term (5-months or 11 months follow-up − pre-test) gain scores for each outcome measure of interest by group, and results from independent *t* tests.
