# Supplementary material for: Effects of Strategy-Based Memory Training for Older Adults: Do Booster Sessions Prompt Long-Term Benefits?
Source: Brain Sci. 2023 Sep 9;13(9):1301. doi: 10.3390/brainsci13091301 (PMC10526930; doi:10.3390/brainsci13091301)
Supplement: Supplementary file 1 [file brainsci-13-01301-s001.zip › brainsci-2542142-supplementary.pdf]

Supplemental materials

Effects of Strategy-Based Memory Training for older adults: Do Booster Sessions prompt Long-Term Benefits?

Table S1. *Summary of studies on strategy-based memory training for older adults including booster sessions.*

Study	Memory training			Booster sessions		Outcomes		Results	
	Content	Schedule	Follow-ups	Content	Schedule	Training gains	Transfer effects	Short-term	Long-term
Ball et al. (2002); Willis et al. (2006)	Participants were taught mnemonic strategies (e.g., organize word lists into meaningful categories, form visual images and mental associations to recall words and texts) and practiced with both laboratory-like memory tasks (eg, recalling a list of nouns, recalling a paragraph), as well as everyday life memory tasks (eg, recalling a shopping list, recalling the details of a prescription label).	10 group-based sessions (60-75 min), twice a week	1-year, 2-year, 3-year, 5-year	Similar to the training sessions	4 group-based sessions (75 min), twice a week, 11 months (right before the 1-year follow-up) and 35 months (right before the 3-year follow-up) from the initial training	Verbal memory ability (HVLT; Rey Auditory-Verbal Learning Test; RBMT-Paragraph Recall test).	Reasoning (letter series, letter sets, and word series), processing speed (useful field of view subscales), everyday functioning (IADL, EPT, TIADL).	Short-term training gains, but no significant transfer effects.	Short-terms gains maintained at 1-year, 2-year and 5-year follow-ups. No additional benefits of booster sessions.
McDougal et al. (2010)	Each session involved: i) relaxation exercise with visualization (20 min); ii) discussion of a class topic (e.g., memory and health, memory functions and mechanisms, factors affecting memory, memory beliefs and aging, external and internal memory strategy use); practice with memory strategies (30 min).	8 group-based sessions, once a week	6-months, 14-months, 26-months	Transitional experience between classroom work on memory improvements and practical applications (boost learning, enhance retention, transfer of memory strategies to everyday life).	4 group-based sessions (120 min), once a week, 5 months (right before the 6-months follow-up) from the initial training	Memory (HVLT; Visuospatial Memory Test–Revised; RBMT), metamemory (MSE; MIA).	General cognitive functioning (MMSE), everyday functioning (DAFS).	Short-term benefits in general and everyday functioning.	No maintenance of benefits.

Aramaki & Yassuda (2011)	Each session involve: i) discussion of a class topic (e.g., memory systems and age-related changes, everyday memory successes), ii) teaching and practice with memory strategies (imagery) to learn word lists or short stories (with fluency tasks between encoding and recall phases)	5 group-based sessions (45 min), twice a week	18 months (pre-test "booster"), about 19 months (post-test "booster")	The same memory training was delivered again.	5 group-based sessions (45 min), twice a week, 18 months after the first memory training	Memory (BCSB-naming (incidental, immediate and delayed recall, verbal fluency; RBMT-immediate and delayed story recall), metamemory (MAC-Q, MSEQ % pictures and stories)	General cognitive functioning (MMSE), executive functioning (Clock drawing), mood (GDS)	Improvements in naming (delayed recall) and self-efficacy (MSEQ, % stories) for the memorization of stories about 19-months after the starting of the first intervention.	Maintenance of short-term benefits. Benefits also in incidental naming recall, delayed story recall, self-efficacy for the memorization of pictures, cognitive failures (MAC-Q) and general cognitive functioning (MMSE).

Notes. HVLT: Hopkins Verbal Learning Test Direct Assessment of Functional Status; RBMT: Rivermead Behavioral Memory test; BCFT: Brief Cognitive Screening Battery; MMSE: Mini-Mental State Examination; IADL: Instrumental Activities of Daily Living scale; TIADL: Timed Instrumental Activities of Daily Living tasks; EPT: Everyday Problems Test; DAFS: Direct Assessment of Functional Status; MIA: Metamemory in Adulthood questionnaire; MSEQ: Memory Self-Efficacy Questionnaire; MAC-Q: Memory Complaint Questionnaire.

Table S2. *Description of the procedure and activities for the strategy-based memory training and the booster sessions.*

Pre-test	Semi-structured interview; MMSE; WL; GL (learning phase); Vocabulary test; GL (recall phase); face-surname associations task; LST; MAC-Q.
**Strategy-based memory training**	
Session 1- "Mental Imagery"	• *Introduction*: welcome and presentations; explanation of the aims of the intervention; reflection on the importance of attitudes towards one' own memory, its functioning, and on the importance of one's commitment to remember effectively.<br>• *Main activities*: the experimenter explains the imagery strategy and how to apply it to remember information, i.e., mentally visualizing to-be-recalled information, also in everyday life; three ad-hoc practical activities were used to allow participants practicing on the vividness of mental images created, a key feature to maximize the learning and remembering of information throughout the use of mental imagery strategy, and learn how to apply both simple and interactive images; two lists of 10 words were then proposed and participants practiced with mental imagery to learn and remember them; group discussion about the usefulness of the imagery strategy and potential issues experienced with it.<br>• *Conclusion*: homework assignments and remind for the next session.<br>• *Homework*: Participants were requested to create and manipulate the features (dimension and movement) of mental images of 3 objects each day, rating the vividness of the mental images created.
Session 2- "Mental Imagery"	• *Introduction*: welcome; discussion on homework assignments; introduction on the main theme of the session.<br>• *Pre-training activity* (music listening)<br>• *Main activities*: the experimenter stresses the importance of the vividness of mental images as a key feature to maximize the learning and remembering of information throughout the use of mental imagery strategy; four lists of 12 words were proposed and participants practiced with mental imagery, either manipulating the dimension of the images, or imagining objects moving, or applying embodied mental imagery techniques (i.e., including proprioceptive, sensorimotor features in the images created, such as visualize oneself interacting with the to-be-imaged objects), to learn and remember them.<br>• *Conclusion*: group discussion about the usefulness of the imagery strategy and potential issues experienced with it; homework assignments and remind for the next session.<br>• *Homework*: participants were given six lists of words (three lists of 5 words, 3 lists of 7 words), each word printed on an index card, and were requested to practice learning and remembering a word list each day applying the mental imagery strategy.
Session 3- "Method of Loci"	• *Introduction*: welcome; discussion on homework assignments; introduction on the main theme of the session.<br>• *Pre-training activity* (music listening)<br>• *Main activities*: the experimenter explains the method of loci and its usefulness for learning and remembering information in everyday life; participants were guided with ad-hoc practical activities to select and memorize a familiar external path with 15 landmarks and did some practical activities with it (mentally moving within their familiar path forward -from the first to the last location and backward -from the last to the first location-) to learn it; the experimenter explained how to use -simple or interactive- images to associate to-be-recalled information successively in each location of one's own path; four lists of 12 words (presented orally by the experimenter) were used to allow participants practicing with the method of loci to learn and remember them; one list of 12 words printed on a sheet of paper was then proposed, and participants were allowed to study it for about 2 min, applying the method of loci, and then remember as many words as they could.<br>• *Conclusion*: group discussion about the usefulness of the method of loci and potential issues experienced with it; homework assignments and remind for the next session.<br>• *Homework*: participants were given three lists of words (10, 12, and 15 words each), each word printed on an index card, and were requested to practice learning and remembering them applying the method of loci.

Session 4- "Face-Surname association"	• *Introduction*: welcome; discussion on homework assignments; introduction on the main theme of the session.<br>• *Pre-training activity* (music listening)<br>• *Main activities*: the experimenter explain how to apply face-surname associations techniques based on mental imagery to successfully remember this information in everyday life; participants were guided with ad-hoc practical activities (face recognition tasks with increasing number of photographs of faces -from 4 to 12-) to select and focus attention to distinctive facial features that make them more memorable; ad-hoc examples and a face-surname recognition task (with 10 photographs of faces coupled with a surname) were then used to allow them practicing with face-surname associations techniques to learn and remember them.<br>• *Conclusion*: group discussion about the usefulness of face-surname associations techniques and potential issues experienced with it; homework assignments and remind for the next session.<br>• *Homework*: participants were given 6 photographs of faces paired with surnames and 6 photographs of faces paired with names, each one printed on a sheet of paper, and were required to learn and remember the name/surname associated with each photograph applying face-name/surname associations techniques.
Session 5- "Method of Loci and Face-Surname association"	• *Introduction*: welcome; discussion on homework assignments; introduction on the main theme of the session.<br>• *Pre-training activity* (music listening)<br>• *Main activities*: participants received additional practice with the method of loci (two lists of 12 words) and face-surname association techniques (a face-surname association task with 10 photographs of faces coupled with a surname).<br>• *Conclusion*: group discussion about the usefulness of the memory strategies acquired throughout the training and their usefulness to remember information in everyday life; participants were asked to give feedback about the engagement and usefulness of the intervention.
Post-test	WL; GL (learning phase); Vocabulary test (filler); GL (recall phase); face-surname associations task; LST; MAC-Q.
**Booster sessions (4 months after the initial memory training completion, for TG1 only)**	
Booster session 1- "Mental Imagery"	• *Introduction*: welcome; explanation of the aims of the booster sessions.<br>• *Pre-training activity* (music listening)<br>• *Main activities:* the experimenter remind participants how to use the mental imagery strategy to successfully learn and remember information in everyday life, with an emphasis on the importance of the vividness of mental images to-be-created; four list of 12 words were used to allow participants practicing with the mental imagery strategy (either manipulating the dimension of the images, or imagining objects moving, or applying embodied mental imagery techniques) to learn and remember them.<br>• *Conclusion*: group discussion about the usefulness of the mental imagery strategy and potential issues experienced with it; homework assignments and remind for the next session.<br>• *Homework*: participants were requested to create and manipulate mental images of 5 objects each day, rating the vividness of the mental images created. Participants were asked to review the familiar path with 15 landmarks learned in the initial training to be used in the next session.
Booster session 2- "Method of Loci"	• *Introduction*: welcome; introduction to the main theme of the session.<br>• *Pre-training activity* (music listening)<br>• *Main activities:* the experimenter remind participants how to use the method of loci to successfully learn and remember information, also in everyday life; participants were guided with ad-hoc practical activities to remind the familiar external path with 15 landmarks selected in the initial training; four lists of 12 words (presented orally by the experimenter) were used to allow participants practicing with the method of loci to learn and remember them.<br>• *Conclusion*: group discussion about the usefulness of the method of loci and potential issues experienced with it; homework assignments and remind for the next session.

	• *Homework*: participants were given four lists of 15 words, each word printed on an index card, and were requested to practice learning and remembering them applying the method of loci.
Booster session 3- "Face-Surname association"	• *Introduction*: welcome; introduction to the main theme of the session.<br>• *Pre-training activity* (music listening)<br>• *Main activities:* the experimenter remind participants how to apply face-surname associations techniques based on mental imagery to successfully remember this information in everyday life; participants were guided with an ad-hoc practical activity (face recognition task) to select and focus attention to distinctive facial features that make them more memorable; ad-hoc examples and two face-surname recognition tasks (with 10 photographs of faces coupled with a surname each) were then used to allow them practicing with face-surname associations techniques to learn and remember them.<br>• *Conclusion*: group discussion about the usefulness of the face-surname associations techniques and potential issues experienced with it; homework assignments; participants were asked to give feedback about the engagement and usefulness of the booster sessions.<br>• *Homework:* participants were given two printed blocks with 6 photographs of faces paired with surnames, and were required to learn and remember the surname associated with each photograph applying face-surname associations techniques.
5-months follow-up	WL; GL (learning phase); Vocabulary test (filler); GL (recall phase); face-surname associations task; LST; MAC-Q.
11-months follow-up	WL; GL (learning phase); Vocabulary test (filler); GL (recall phase); face-surname associations task; LST; MAC-Q.

Notes. WL: word list recall task; GL: grocery list recall task; LST: Listening Span Test; MAC-Q: Memory Complaint Questionnaire.

Table S3. *Descriptive statistics for the short-term (post-test – pre-test) and long-term (5-months or 11 months follow-up – pre-test) gain scores for each outcome measure of interest by group, and results from independent t tests.*

	Short-term benefits				Long-term benefits (5-months follow-up)				Long-term benefits (11-months follow-up)			
	TG1	TG2			TG1	TG2			TG1	TG2		
	M (SD)	*M (SD)*	$t_{(1,31)}$	*p*	*M (SD)*	*M (SD)*	$t_{(1,24)}$	*p*	*M (SD)*	*M (SD)*	$t_{(1,22)}$	*p*
Word List	1.24 (2.016)	0.69 (2.089)	0.767	.449	1.36 (2.292)	1.53 (1.457)	-.216	.832	1.55 (1.916)	1.46 (1.664)	.436	.910
Face-surname associations task	1.25 (1.693)	0.81 (1.940)	0.680	.502	0.91 (2.468)	1.60 (2.197)	-0.752	.459	1.3 (1.567)	1.69 (1.750)	-.557	.583
Gocery list	0.76 (2.705)	1.13 (2.754)	-.379	.707	1.36 (3.668)	-0.07 (1.907)	1.296	.207	1.09 (2.256)	-0.15 (1.463)	1.628	.118
Listening Span Test	1.71 (3.478)	1.56 (2.449)	0.136	.893	1.91 (2.948)	1.27 (2.463)	0.605	.551	1.18 (1.722)	1.62 (3.525)	-0.392	.700
Memory Complaint Questionnaire	-1.06 (3.288)	-0.44 (3.949)	-0.492	.626	-.109 (3.419)	-0.07 (3.990)	-0.686	.449	0.09 (1.921)	-1.23 (4.106)	0.978	.339
